# Cutaneous Sarcoidosis of a 53-Year-Old Female: A Case Report

**DOI:** 10.7759/cureus.19351

**Published:** 2021-11-08

**Authors:** Ali Alghamdi, Nadia Mazraani, Salman A Thabet, Basel Saeed Alghamdi, Maha Hanawi, Hatim Almaghraby, Hassan F Huwait

**Affiliations:** 1 Internal Medicine, King Saud Bin Abdulaziz University for Health Sciences, College of Medicine-Western Region, Ministry of National Guard Health Affairs, King Abdullah International Medical Research Center, Jeddah, SAU; 2 Family Medicine, King Saud Bin Abdulaziz University for Health Sciences, College of Medicine-Western Region, Ministry of National Guard Health Affairs, Jeddah, SAU; 3 Internal Medicine, King Saud Bin Abdulaziz University for Health Sciences, College of Medicine-Western Region, Ministry of National Guard Health Affairs, Jeddah, SAU; 4 Plastic Surgery, King Abdullah Medical City, Jeddah, SAU; 5 Pathology, Ministry of National Guard Health Affairs, King Abdullah International Medical Research Center, Jeddah, SAU; 6 Dermatopathology, Umm Al-Qura University, Mecca, SAU

**Keywords:** cutaneous, sarcoidosis, non-caseating, granuloma, epithelioid

## Abstract

Sarcoidosis is a systemic disease of an unknown cause that affects multiple organs, most commonly lungs, intrathoracic lymph nodes, eyes, and skin, which accounts between 20% and 25%. However, cutaneous sarcoidosis can present without any systemic involvement in 25% of cases. We present a case of a 53-year-old female patient with cutaneous sarcoidosis with no lung involvement. The patient presented to the family medicine department with non-itchy, tender, erythematous papules occurring at the dorsal part of the hands and the right foot for three months. Skin punch biopsy demonstrated multiple dermal-based nodules consisting of non-necrotizing granulomata. Serum angiotensin-converting enzyme level and a chest radiograph were normal and not consistent with pulmonary sarcoidosis. There are different cutaneous manifestations of cutaneous sarcoidosis and early identification helps in early intervention.

## Introduction

Sarcoidosis is a chronic granulomatous disease that affects multiple organ systems in the body. It is characterized by the formation of epithelioid and non-caseating granulomas. The most common organs that are affected by sarcoidosis are the lungs, intrathoracic lymph nodes, eyes, and skin [1]. The prevalence of sarcoidosis per 100,000 cases ranges from 0.04 to 64 cases [1]. Although the exact causes of sarcoidosis are unknown, certain factors may play a role in the pathogenesis. Among these are genetic susceptibility with the human leukocyte antigen (HLA) genes being implicated, immune system weakness, and environmental factors [1-4]. As a result, its incidence differs worldwide due to different genetics and variable geographical areas with distinct seasons and infections exposure. The highest prevalence of sarcoidosis worldwide is observed in African-Americans and Scandinavians [2]. In addition, females are more prone to get sarcoidosis than males [1,3]. Management of sarcoidosis is done by a multidisciplinary team. Not all patients need treatment and the disease may resolve spontaneously in some cases. The mainstay treatment in sarcoidosis is immunosuppressive therapy such as corticosteroids either topical or systemic options, methotrexate, and tumor necrosis factor-alpha inhibitors adalimumab and infliximab [4].

## Case presentation

**Figure 1:**
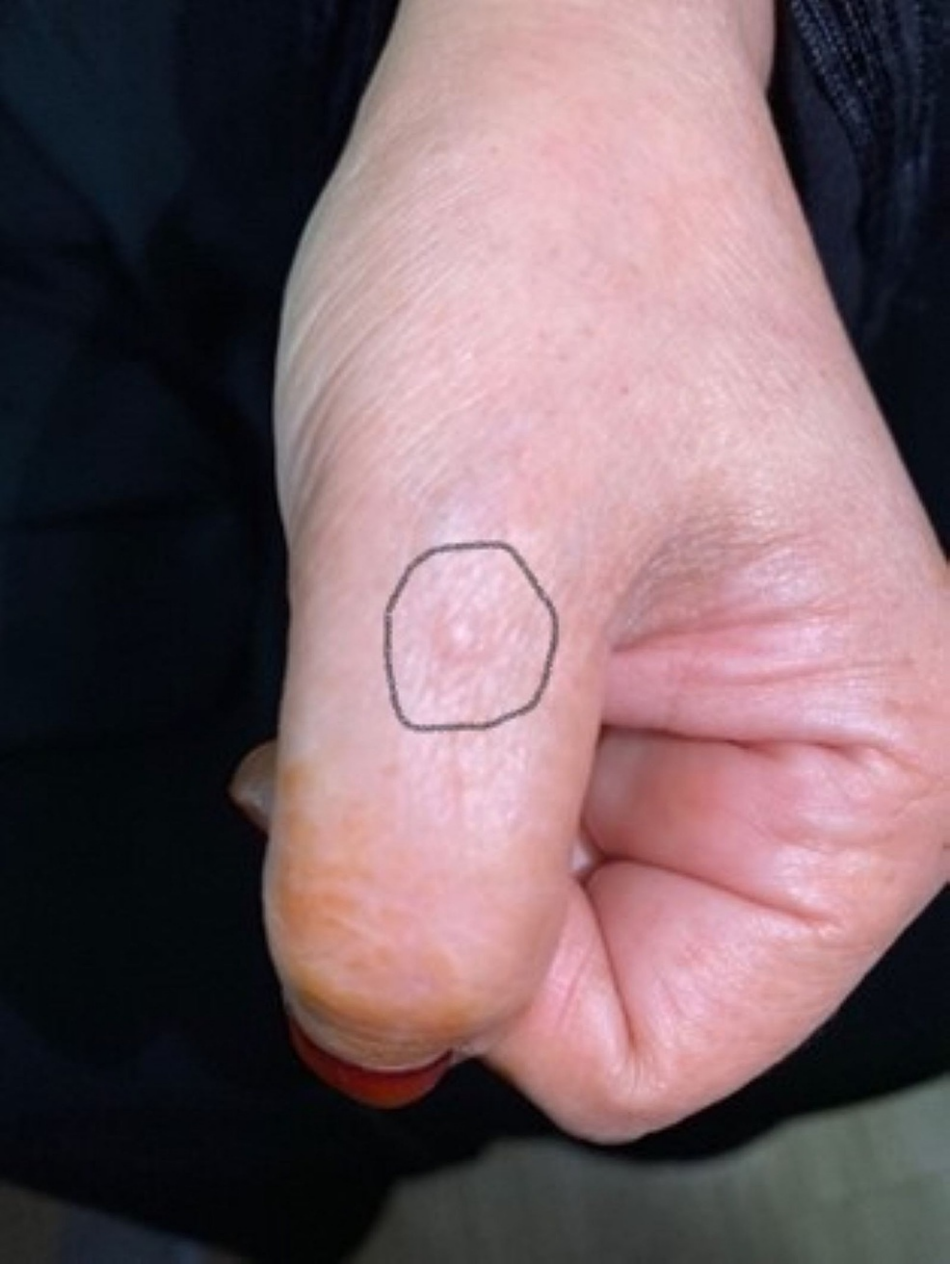
Skin lesion on the thumb Figure shows skin-colored papules on the thumb, which are indurated and tender on palpation.

**Figure 2:**
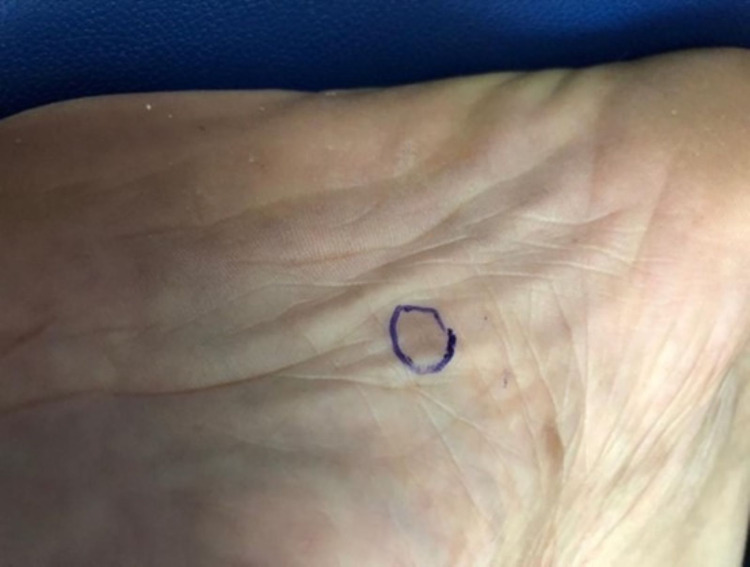
Skin lesion on the sole Figure shows skin-colored papules on the sole of the foot, all of which are indurated and tender on palpation.

**Figure 3:**
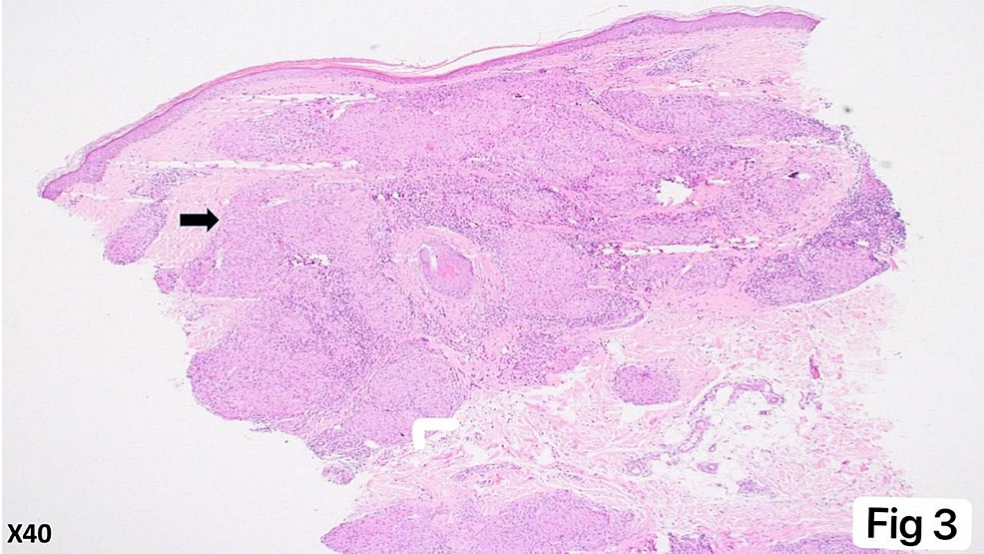
Low-power view of the skin biopsy Low-power view demonstrates dermal-based relatively circumscribed nodular infiltrates (see arrow) involving the papillary and reticular dermis.  The infiltrate surrounds the hair follicle and adnexal structures without directly involving them (H&E, x40 magnification).

**Figure 4:**
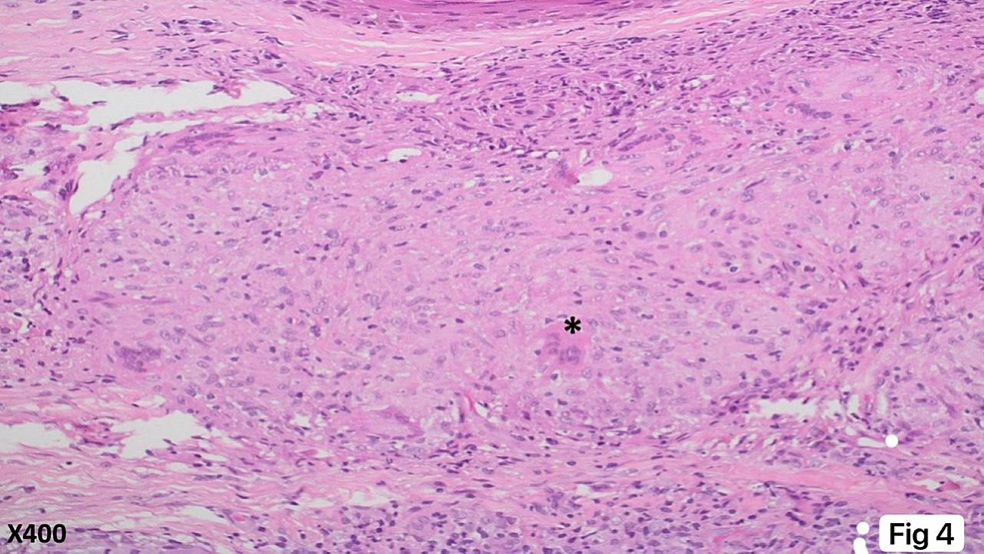
High-power view of the skin biopsy High-power view demonstrates that the infiltrates consist of epithelioid histiocytes and scattered multi-nucleated giant cells (asterisk). This type of granuloma (sarcoid-like) shows lack of necrosis and lack of noticeable or sparse lymphocytic infiltrates (H&E, x400).

A 53-year-old female patient presented to the family medicine department with “multiple small hard erythematous tender papules with no secondary changes over the dorsal aspect of the hands and right sole for three months increasing with time" (see Figures 1, 2). Also, she had a history of chronic low blood pressure lasting for four years and mild degenerative changes in L4-L5 vertebrae with bilateral pars defect and no significant spondylolisthesis that predated her cutaneous complaint. She was referred to the orthopedic department regarding her back complaint and refused the operation. In addition, she was complaining of shortness of breath and coughing. There were no significant systemic and constitutional symptoms, nor a history of trauma. General physical and systemic examination was normal. Dermatological history was not significant except for seborrheic dermatitis and hair loss years ago. Dermatological examination revealed multiple, well-demarcated, small, hard, erythematous, tender, non-itchy papules over the dorsal part of the hands and foot, measuring 3-5 mm, without secondary changes (Figures 1, 2). Hematological and biochemical investigations were done and showed elevated C-reactive protein and erythrocytes sedimentation rate 15 milligrams/liter, and 34 millimeters (mm), respectively. Serum angiotensin-converting enzyme (ACE) level was normal at 38 IU/ml (normal range is 18-67 IU/ml). Chest radiograph was unremarkable for cardio mediastinal shadow, and both lung fields were clear and not consistent with pulmonary sarcoidosis changes. The pulmonary function test was normal. A 5-mm skin punch biopsy demonstrated multiple dermal-based nodules consisting of non-necrotizing granulomata. The granulomata were surrounded by a meager cuff of lymphocytes and consisted of epithelioid macrophages and multinucleated giant cells (see Figures 3, 4). Special stains for acid-fast bacilli and fungi were negative. Our dermatologist prescribed for her mometasone furoate 0.1% cream, 30 g twice daily then 2 times per week for 8 weeks, and tacrolimus 0.1% ointment (LASA) twice daily. The lesions in the hands resolved completely after one month of the treatment course; however, the lesion in the right foot reduced but did not disappear.

## Discussion

The skin is the second most common organ affected by sarcoidosis. According to PubMed published data by Karolyn A. Wanat, 25-30% of sarcoidosis cases had skin manifestations. The highest incidence of cutaneous sarcoidosis occurs in Black American females [5]. Cutaneous sarcoidosis lesions could occur in any area of the skin but they are more pronounced in previously manipulated areas as in tattoos and scars [5]. Different primary lesions such as macules, patches, papules, and plaques may appear, as well. The neck, upper back, and trunk are the most commonly involved areas [3]. Skin lesions of sarcoidosis are divided into specific and non-specific lesions. Biopsy of specific lesions revealed granulomas, whereas the non-specific ones demonstrate inflammation without granulomatous formation [1,5]. Our case is a 53-year-old middle-eastern female with features of a specific type presented with multiple hard, tender, erythematous papules on the hands and feet.

Sarcoidosis diagnosis is based on clinical manifestations by appropriate history taking, physical examination, radiological and histopathological findings, and, finally, exclusion of other granulomatous diseases. The clinical presentation of sarcoidosis is variable depending on the affected organs and the severity of the disease. Many patients are asymptomatic in the initial stages, and they are diagnosed incidentally based on a chest radiograph. On the other hand, up to half of the patients complain initially from respiratory symptoms such as shortness of breath and dry cough, and one-third of the patients may present with constitutional symptoms such as fever and significant weight loss [2]. Radiological findings are manifested as bilateral hilar lymphadenopathy and parenchymal nodules on chest radiograph. In addition, a biopsy is obtained to confirm the diagnosis by histopathological findings as non-caseating granulomas [1-4]. Our case complained of shortness of breath upon presentation to the family department, yet there were no abnormal findings on the chest radiograph. Moreover, the patient was not complaining of any constitutional symptoms or lymphadenopathy involvement. Histopathology report revealed granulomatous dermatitis with sarcoid-type granuloma, and focal polarizable foreign material was recognized.

Serum ACE, which is derived from epithelioid cells of the granulomas, reflects the granuloma load in the patient and is usually elevated in around 60% of patients. Serum ACE levels are neither diagnostic nor predictors of systemic involvement, yet they may be useful for predicting disease progression, yet our patient presented with a normal level of serum ACE [6].

## Conclusions

Sarcoidosis is a disease with multiple organs' involvement. Cutaneous manifestations of sarcoidosis are different and non-specific. The diagnosis is confirmed by the presence of non-caseating epithelioid granulomas in histological findings. Topical and systemic corticosteroid and other immunosuppressive medications such as methotrexate, adalimumab, and infliximab are used in cutaneous sarcoidosis management. 

## References

[1] Salah S, Abad S, Monnet D, Brézin AP (2018). Sarcoidosis. J Fr Ophtalmol.

[2] Sève P, Pacheco Y, Durupt F (2021). Sarcoidosis: a clinical overview from symptoms to diagnosis. Cells.

[3] Abed Dickson M, Hernández BA, Marciano S, Mazzuoccolo LD (2021). Prevalence and characteristics of cutaneous sarcoidosis in Argentina. Int J Womens Dermatol.

[4] Llanos O, Hamzeh N (2019). Sarcoidosis. Med Clin North Am.

[5] Caplan A, Rosenbach M, Imadojemu S (2020). Cutaneous sarcoidosis. Semin Respir Crit Care Med.

[6] Kraaijvanger R, Janssen Bonás M, Vorselaars ADM, Veltkamp M (2020). Biomarkers in the diagnosis and prognosis of sarcoidosis: current use and future prospects. Front Immunol.

